# Baicalein, an active component of *Scutellaria baicalensis* Georgi, prevents lysophosphatidylcholine-induced cardiac injury by reducing reactive oxygen species production, calcium overload and apoptosis via MAPK pathways

**DOI:** 10.1186/1472-6882-14-233

**Published:** 2014-07-09

**Authors:** Huai-Min Chen, Jong-Hau Hsu, Shu-Fen Liou, Tsan-Ju Chen, Li-Ying Chen, Chaw-Chi Chiu, Jwu-Lai Yeh

**Affiliations:** 1 Graduate Institute of Medicine, College of Medicine, Kaohsiung Medical University, Kaohsiung, Taiwan; 2 Division of Cardiovascular Surgery, Department of Surgery, Kaohsiung Medical University Hospital, Kaohsiung, Taiwan; 3 Department of Paediatrics, Kaohsiung Medical University Hospital, Kaohsiung, Taiwan; 4 Department of Paediatrics, Faculty of Medicine, College of Medicine, Kaohsiung Medical University, Kaohsiung, Taiwan; 5 Department of Pharmacy, Chia-Nan University of Pharmacy and Science, Tainan, Taiwan; 6 Department of Physiology, College of Medicine, Kaohsiung Medical University, 100 Shih-Chuan 1st Road, Kaohsiung 807, Taiwan; 7 Department and Graduate Institute of Pharmacology, College of Medicine, Kaohsiung Medical University, 100 Shih-Chuan 1st Road, Kaohsiung 807, Taiwan

**Keywords:** Baicalein, Lysophosphatidylcholine, Apoptosis, Reactive oxygen species, Calcium

## Abstract

**Background:**

Lysophosphatidylcholine (lysoPC), a metabolite from membrane phospholipids, accumulates in the ischemic myocardium and plays an important role in the development of myocardial dysfunction ventricular arrhythmia. In this study, we investigated if baicalein, a major component of Huang Qui, can protect against lysoPC-induced cytotoxicity in rat H9c2 embryonic cardiomyocytes.

**Methods:**

Cell viability was detected by the MTT assay; ROS levels were assessed using DCFH-DA; and intracellular free calcium concentrations were assayed by spectrofluorophotometer. Cell apoptosis and necrosis were evaluated by the flow cytometry assay and Hoechst staining. Mitogen-Activated Protein Kinases (MAPKs), which included the ERK, JNK, and p38, and the apoptotic mechanisms including Bcl-2/Bax, caspase-3, caspase-9 and cytochrome *c* pathways were examined by Western blot analysis. The activation of MAPKs was examined by enzyme-linked immunosorbent assay.

**Results:**

We found that lysoPC induced death and apoptosis of H9c2 cells in a dose-dependent manner. Baicalein could prevent lysoPC-induced cell death, production of reactive oxygen species (ROS), and increase of intracellular calcium concentration in H9c2 cardiomyoctes. In addition, baicalein also inhibited lysoPC-induced apoptosis, with associated decreased pro-apoptotic Bax protein, increased anti-apoptotic Bcl-2 protein, resulting in an increase in the Bcl-2/Bax ratio. Finally, baicalein attenuated lysoPC-induced the expression of cytochrome *c*, casapase-3, casapase-9, and the phosphorylations of ERK1/2, JNK, and p38. LysoPC-induced ERK1/2, JNK, and p38 activations were inhibited by baicalein.

**Conclusions:**

Baicalein protects cardiomyocytes from lysoPC-induced apoptosis by reducing ROS production, inhibition of calcium overload, and deactivations of MAPK signaling pathways.

## Background

Lysophosphatidylcholine (lysoPC) is generated by the phospholipase A2-dependent hydrolysis of phosphatidylcholine in the membranes of injured cardiomyocytes and may cause deleterious effects on cardiac function during cardiac ischemia [1]. In addition, lysoPC can induce the increase of the intracellular Ca^2+^ concentration ([Ca^2+^]i) and plays an important role in triggering onset of arrhythmias in cardiac ischemia [2]. Besides, lysoPC triggers apoptosis of vascular smooth muscle cells and endothelial cells by altering intracellular calcium signaling [3,4]. LysoPC also is one of the key components accumulating in the atherosclerotic lesions, and participate in the initiation and/or progression of atherosclerosis [3-5].

Oxidative stress in cardiomyocytes plays an important role in the pathogenesis of both heart failure and ischemic reperfusion injury. The reactive oxygen species (ROS) can cause severe myocardium damage because the cardiac system carries lesser superoxide dismutase, glutathione, and catalase to remove the superoxidative toxic substances [6]. Huang Qui (*Scutellaria baicalensis* Gerorgi) is a raw material of traditional Chinese Pharmacopoeia commonly used to improve the physique, and resist inflammation and microorganisms. The compositions of baicalein, wagonin, skullcap-flavone I & II in Huang Qui all have anti-oxidative effects, so Huang Qui has been implicated in the prevention of the ischemia-reperfusion injuries and in the reduction of the ROS productions [7,8]. Baicalein (5,6,7-trihydroxy-2-phenyl-4H-1-benzopyran-4-one) is a major bioactive flavones constituent of Huang Qui, and shows a variety of biological activities, including antioxidant, anti-inflammatory, antithrombotic, antiviral, and anticancer activities. In deed, in previous studies baicalein has been found to effectively lower ROS-induced cells death in chicken fetal cardiomyocytes in the setting of hypoxic injury [7-9].

Baicalein has previously been shown to possess not only anti-apoptotic effects on cardiomyocytes but also anti-proliferative effects in VSMCs [7-9]. However, it is unclear whether this agent may protect cardiomyocytes from lysoPC-induced cytotoxicity, and if so, what the underlying mechanisms are. Therefore, this study was undertaken to determine the protective effects and mechanisms of baicalein in lysoPC-induced cytotoxicity in H9c2 cardiomyocytes.

## Methods

Lysophosphatidylcholine, baicalein, fura-2/AM, β-actin antibody and 3-[4,5-dimethylthiazol-2-yl]-2,5-diphenyl tetrazolium bromide (MTT) were obtained from Sigma Aldrich Chemical Company (St. Louis, MO, USA). 2′,7′-Dichlorodihydrofluorescein diacetate (DCFH-DA) was purchased from Molecular Probes (Eugene, OR). Antibodies to Bcl-2, Bax, ERK1/2, JNK and phosphorylated JNK were obtained from Upstate Biotechnology (Lake Placid, NY, USA) while antibodies of p38, phosphorylated p38 and cytochrome *c* were obtained from Santa Cruz Biotech (Santa Cruz, CA, USA). Antibodies to cleaved caspase-3, cleaved caspase-9, and phosphorylated ERK1/2 were obtained from Cell Signaling Technology (Beverly MA, USA). Dulbecco’s modified Eagle’s medium (DMEM), fetal bovine serum (FBS), penicillin, streptomycin, and all other tissue culture reagents were obtained from GIBCO BRL Life Technologies (Grand Island, NY, USA). In the experiments, baicalein was prepared by dissolving with DMSO. Dilutions were made in phosphate-buffered saline (PBS) and filtered through a 0.22 μM syringe filter.

### Cell culture

The rat ventricular myocardial cell line H9c2 was obtained from the American Type Culture Collection (ATCC, Rockville, MD, USA) and cultured in Dulbecco’s Modified Eagle Medium (DMEM) (GIBCO BRL Life Technologies, NY, USA) supplemented with 10% fetal bovine serum (Kibbutz Haemek, Israel) containing 100 U/ml of penicillin G, 100 μg/ml streptomycin and 0.25 mg/ml amphotericin B in a humidified atmosphere containing 5% CO_2_ at 37°C. Before experimental intervention, confluent-cultured cells were serum-starved for 24 h in DMEM supplemented with 0.1% fetal bovine serum.

### Determination of cells viability

To assess H9c2 cell viability, MTT assay was performed according to the manufacturer’s instructions. After the experiments, MTT (0.5 mg/ml) was added in the medium for 4 h. The culture medium was removed and the cells were dissolved in isopropanol and shaken for 10 min. The amount of MTT formazan was quantified at absorbance of 540 nm and 630 nm using an ELISA reader (DYNEX Technologies, Germany).

### Measurement of intracellular free calcium concentration

Intracellular calcium concentration ([Ca^2+^]_i_) was measured as we previously described [10]. Trypsinized cells (1 × 10^6^ cells/ml) were loaded with 2 μM of the ester form of fura-2 (fura-2/acetoxy methyl) for 30 min at 25°C in DMEM. After loading, the cells were kept in a balanced salt solution (BSS, mM: 140 NaCl, 5 KCl, 1 MgCl_2_, 2 CaCl_2_, 10 HEPES, 5 glucose, pH 7.4). Fura-2 fluorescence measurements were performed in a water-jacketed cuvette (25°C) with continuous stirring. The cuvette contained 1 ml of BSS and 5 × 10^5^ cells. The H9c2 cells were incubated in Ca^2+^-containing buffer with various concentrations of baicalein for 5 min. Fluorescence was monitored with a Shimadzu RF-5301PC spectrofluorophotometer (Shimadzu, Kyoto, Japan) by recording excitation signals at 340 and 380 nm, and emission signals at 510 nm in two-second intervals.

### Measurement of intracellular ROS production

Levels of intracellular O_2_^−^ and H_2_O_2_ were assessed spectrofluorometrically by oxidation of specific probes: DCFH-DA. Dye loading was performed by incubating the cardiomyocytes with 10 μM DCFH-DA for 30 min at 37°C, and the fluorescence intensity of the cells was determined using fluorescent microscope and fluorescence-activated cell sorting (FACS) analysis.

### Hoechst 33342 staining

Apoptotic cells were confirmed by Hoechst 33342 staining. The cells showing nuclear fragmentation and chromatin condensation in Hoechst staining were categorized as apoptotic cells [11]. After drug pretreatment for 24 h, the cultured H9c2 cells were exposed to 10 μM lysoPC. Then, cells were washed with 1X Hank’s balance salt solution (HBSS) and stained with 10 μg/ml of Hoechst 33342 for 60 min. The nuclear morphology of the cells was visualized using a fluorescence microscope (Zeiss Axioskop 2 plus, Japan).

### Annexin V/propidium iodide staining

Quantitative assessment of apoptotic cells was determined by flow cytometry using the Annexin V-conjugated Alexa Fluor 488 Apoptosis Detection Kit following the manufacturer’s instructions. The apoptotic and necrotic cells from the same samples were quantified using quantitative FACS analysis. This method utilizes the binding of FITC-labeled Annexin V to phosphatidylserine in the cell membrane that surfaces only during the early phase of apoptosis, indicating the loss of cell membrane phospholipid asymmetry. However, the apoptotic cells with intact cell membranes do not stain with the propidium iodide. By utilizing the morphological changes that occur in both apoptotic and necrotic cells, the samples were stained simultaneously with Annexin V-FITC and propidium iodide. The samples were then subjected to flow cytometric analyses to detect the percentage of apoptotic (FITC-stained cells) and necrotic cells (PI-stained cells) in a given population. A minimum of 10,000 cells were maintained for all the samples. The samples were analyzed by a Coulter Epics XL-MCL (Beckman Coulter, USA).

### Western blotting

After treatment with the indicated agents, cells were washed twice with cold PBS and then cells were harvested. Total cell extracts were prepared in lysis buffer [20 mM Tris–HCl (pH 7.5), 1 mM dithiothreitol (DTT), 5 mM EGTA, 2 mM EDTA, 0.5 mM PMSF, 20 μM leupeptin, and 20 μM aprotinin]. The cell lysate was centrifuged at 15,000 g for 30 min, and the supernatant fraction was collected for Western blot. An equivalent amount of protein was resolved by SDS-polyacrylamide gel electrophoresis (PAGE) (10-14%) and transferred to polyvinylidene difluoride membranes. After blocking for 1 h in 5% non-fat dry milk in Tris-buffered saline, the membrane was incubated with the desired primary antibody for 2 h. The membrane was then treated with appropriate horseradish peroxidase-conjugated secondary antibody (diluted 1:1000), and the immunoreactive bands were detected with enhanced chemiluminescence reagents (PerkinElmer Life and Analytical Sciences).

### Measuring MAPK activity

H9c2 cells were pretreated with baicalein (0.1 to 10 μM) for 1 h followed by incubation with lysoPC (10 μM) for 10 min. Phosphorylated protein levels of ERK1/2, JNK, and p38 MAPK were determined using cell based ELISA kit (RayBio® Cell-Based ERK1/2, JNK, p38 MAPK phosphorylation ELISA Sampler Kit, Ray Biotech Inc., Norcross, GA, USA) as per manufacturer’s instructions.

### Statistical analysis

Data are expressed as means ± SEM. Statistical differences were estimated by one-way analysis of variance (ANOVA) followed by Dunnett’s test. A value of *P* < 0.05 was considered significant.

## Results

### Baicalein inhibited cell death induced by lysoPC

**Figure 1:**
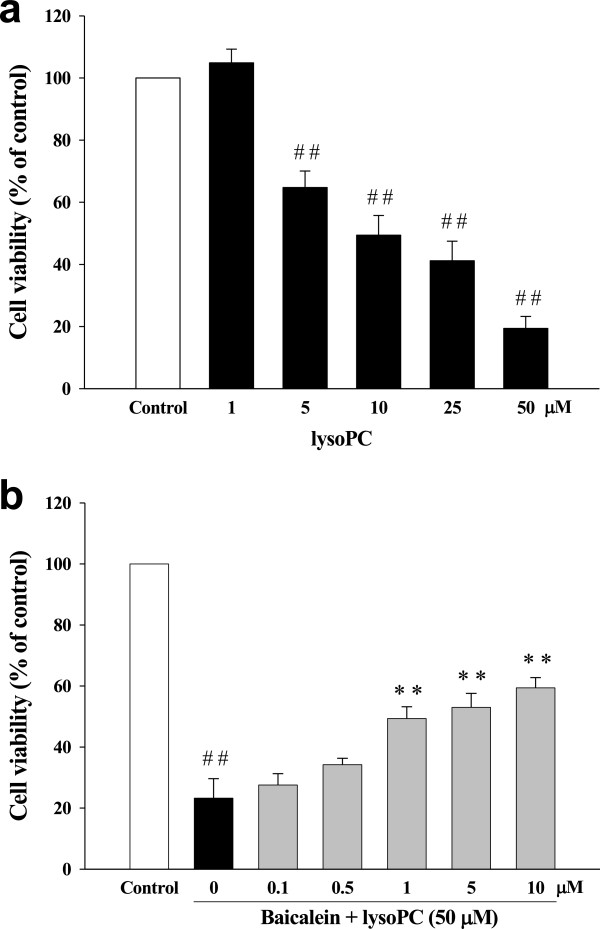
**Protective effects of baicalein on lysophosphatidylcholine (lysoPC)-induced cell death of rat H9c2 cells. (a)** H9c2 cells were treated with lysoPC for 24 h. LysoPC (1 to 50 μM) dose-dependent decreased cell viability. **(b)** Baicalein attenuated lysoPC (50 μM)-induced cell death in a dose-dependent manner. Values represent mean ± S.E.M, n = 9. Control: H9c2 cells were placed in serum-free medium. ^##^*P* < 0.01, versus control group; **P < 0.01 versus cells exposed to lysoPC alone.

To determine the dose–response effects of lysoPC on death of rat H9c2 cells, a series of experiments was performed using the MTT assay in different concentrations of lysoPC. Figure 1a shows lysoPC (5 to 50 μM) caused significant cell death. Baicalein pretreatment of H9c2 cells attenuated lysoPC-induced cell death in a concentration-dependent manner (Figure 1b).

### Baicalein decreased lysoPC-induced ROS production

**Figure 2:**
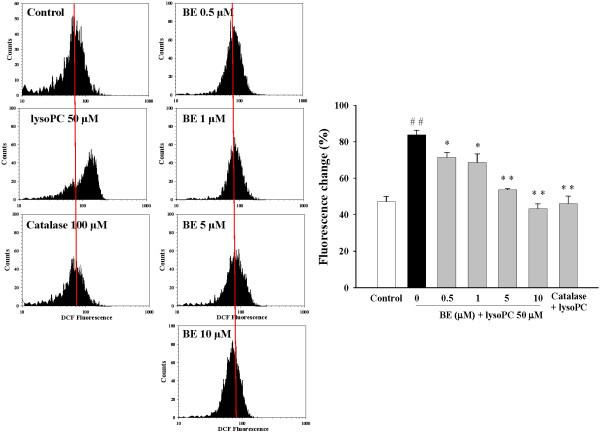
**Inhibitory effects of baicalein (BE) and catalase (100 μM) on lysoPC-induced ROS production in H9c2 cells.** Treatment with 50 μM lysoPC for 20 min increased the production of ROS, BE significantly reduced ROS production in a dose-dependent manner, as revealed by DCFH-DA staining. Values represent mean ± S.E.M of three independent experiments, which triplicate determinations in each experiment. ^##^*P* < 0.01 versus control; *P < 0.05, **P < 0.01 versus cells exposed to lysoPC alone.

Oxidation of intracellular DCFH-DA to fluorescent DCF was observed in lysoPC-treated H9c2 cells as shown by the greater right shift of the mean fluorescence value, which was higher in lysoPC (50 μM)-treated cells than untreated cells, indicating the stimulation of ROS production. Figure 2 shows that pretreatment with baicalein significantly attenuated the production of ROS induced by lysoPC in a concentration-dependent manner. Catalase (100 μM) reduction of lysoPC-induced ROS production to the control level was used as a positive control.

### Baicalein attenuated lysoPC-induced Ca^2+^ influx

**Figure 3:**
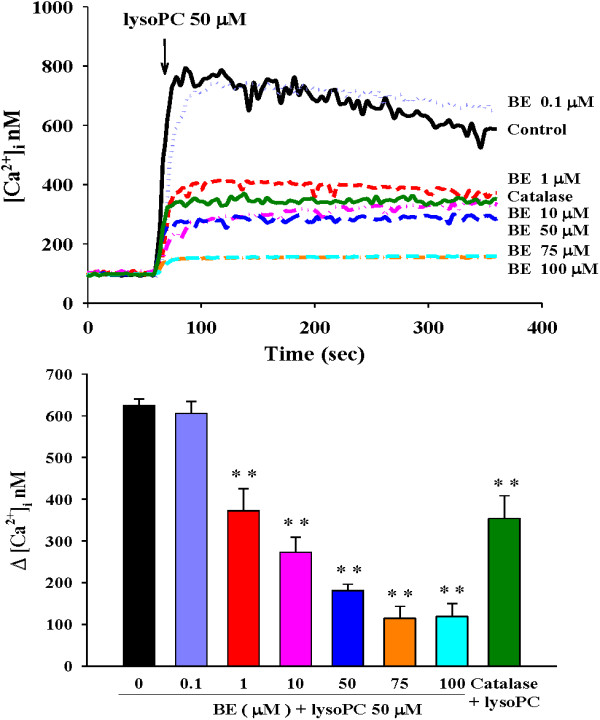
**Effects of baicalein (BE) on lysoPC-induced Ca**^**2+**^**responses.** Typical curves of H9c2 cells which were pretreated by BE (0.1 to 100 μM) or catalase (100 μM) 5 min before stimulation with 50 μM lysoPC in calcium-containing buffer. The lower panel indicates the influence of BE and catalase on the variations in [Ca^2+^]_i_ measured at the peak and expressed as [Ca^2+^]_i_ (i.e., after subtraction of the respective baseline values). Pretreatment with BE and catalase had inhibitory effects on increased [Ca^2+^]_i_ induced by lysoPC. Arrows indicate the time points of adding lysoPC, n = 4–6. **P < 0.01 versus cells exposed to lysoPC alone.

LysoPC caused Ca^2+^ influx in H9c2 cells as shown by increased [Ca^2+^]i. However, these effects of lysoPC (50 μM) were blunted by baicalein pretreatment in a dose-dependent manner (Figure 3). We found that the IC50 value of baicalein was 0.69 μM. Similarly, catalase (100 μM) also had significant inhibitory effect on lysoPC-induced Ca^2+^ influx (Figure 3).

### Baicalein protects H9c2 cells from lysoPC-induced apoptosis

**Figure 4:**
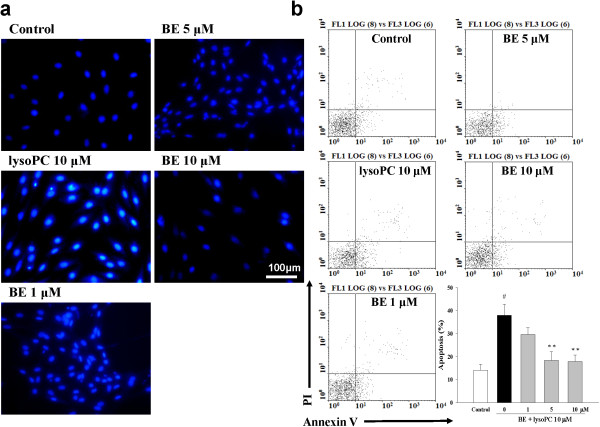
**Effects of baicalein (BE) on lysoPC-induced apoptosis.** H9c2 cells were pretreated with or without BE before being stimulated by lysoPC (10 μM). **(a)** Fluorescence photomicrographs of cells stained with Hoechst 33342, showing nucleus exhibiting brightly stained condensed chromatin, indicating typical morphological changes of apoptosis. **(b)** Analysis of apoptotic cells defined by annexin V-positive cells. The effects of BE (1, 5, and 10 μM) on lysoPC-induced apoptosis of H9c2 cells were determined by flow cytometry. Quantitative bar graphs showing apoptotic cell counts determined by flow cytometry. Each value represents the mean ± SEM of three independent experiments, with triplicate determinations in each experiment. ^#^*P* < 0.05 compared with control; **P < 0.01 versus cells exposed to lysoPC alone.

To further examine the effect of baicalein on apoptosis, we assessed the protective effect of baicalein on lysoPC-induced apoptosis by Hoechst 33342 staining (Figure 4a). H9c2 cells with condensed and fragmented nuclei and apoptotic bodies were seen when exposed to 10 μM lysoPC for 24 h. Pretreatment with 10 μM baicalein for 24 h resulted in a significant decrease in the number of Hoechst-positive apoptotic cells (Figure 4a). A quantitative evaluation of apoptosis was also made using Annexin V-conjugated Alexa Fluor 488 Apoptosis Detection Kit (Figures 4b and c). Annexin V-conjugated FITC specifically binds to phosphatidylserine residues of apoptotic cells. LysoPC-induced apoptosis in H9c2 cells, while pretreatment with baicalein (1 to 10 μM) had similar protective effects to reduce the percentage of apoptosis.

### Baicalein attenuated lysoPC-induced apoptosis through inactivation of the mitochondrial pathway

**Figure 5:**
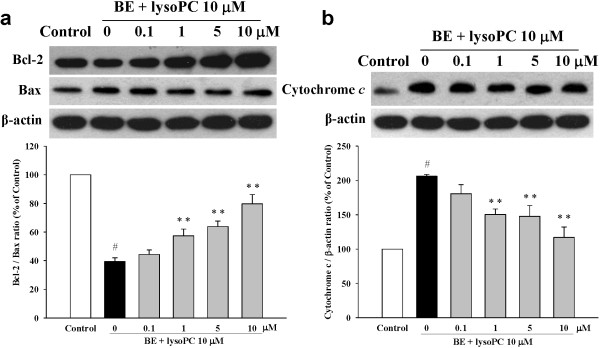
**Effects of baicalein (BE) on pro-apoptotic proteins. (a)** Cells were pretreated with BE (0.1 to 10 μM) for 1 h followed by incubation with lysoPC (10 μM) for 24 h. The expression levels of Bcl-2 protein and Bax protein are shown in the upper panel. Quantitative analysis on the Bcl-2/Bax ratio, an index of antiapoptotic trend, is shown in the lower panel. **(b)** Inhibitory effects of BE on cytochrome *c* release in lysoPC-induced H9c2 cells for 24 h. Each value represents the mean ± S.E.M, n = 3–4. ^#^*P* < 0.05 versus control; **P < 0.01 versus cells exposed to lysoPC alone.

To investigate the mitochondrial apoptotic events involved in baicalein-reduced apoptosis, we first analyzed the changes of Bcl-2 family members, which included proapoptotic protein (Bax) and anti-apoptotic protein (Bcl-2), two important mediators that trigger mitochondrial depolarization in the process of intrinsic apoptosis. Our results showed that lysoPC down-regulated Bcl-2 expression and up-regulated Bax expression, and pretreatment with baicalein increased the Bcl-2 protein level and decreased the Bax level (Figure 5a, upper panel), together resulting in an increase in the Bcl-2/Bax ratio (Figure 5a, low panel).

**Figure 6:**
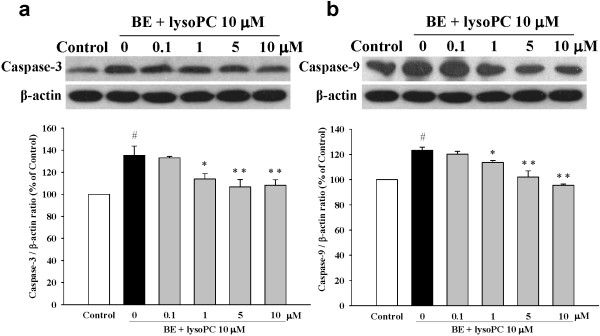
**Inhibitory effects of baicalein (BE) on lysoPC-induced expression of caspase-3 (a) and caspase-9 (b).** Cells were pretreated with BE (0.1 to 10 μM) for 1 h followed by incubation with lysoPC (10 μM) for 24 h. Each value represents the mean ± S.E.M, n = 3–4. ^#^*P* < 0.05 versus control; *P < 0.05, **P < 0.01 versus cells exposed to lysoPC alone.

Western blot analysis also showed that treatment of H9c2 cells with lysoPC increased cytochrome *c* level in the cytosol. When the H9c2 cells were treated with 10 μM lysoPC in the presence of 1 to 10 μM baicalein for 24 h, the band was attenuated remarkably (Figure 5b), which suggested that treatment with baicalein can suppress mitochondrial pore transition and reduce the cytochrome *c* release into the cytosol.We further determined potential mechanisms underlying baicalein’s anti-apoptotic effects by examining caspase-3 and caspase-9, two down-stream mediators of mitochondrial apoptosis pathway. LysoPC significantly increased both protein expression and activity of caspase-3 and caspase-9 in H9c2 cells (Figures 6a and b). However, these effects were attenuated by baicalein pre-treatment in a dose-dependent manner.

### Baicalein mediated protection from apoptosis via MAPKs-dependent signaling pathway

**Figure 7:**
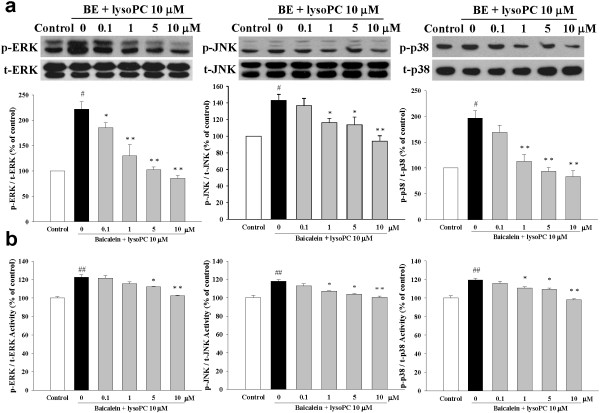
**Inhibitory effects of baicalein (BE) on lysoPC-induced phosphorylation of ERK, JNK and MAPK.** Cells were pretreated with BE (0.1 to 10 μM) for 1 h followed by incubation with lysoPC (10 μM) for 10 min. (**a**, up panel) Baicalein attenuated lysoPC-induced phosphorylation of ERK1/2, JNK and p38, as shown by Western blotting. (**b**, down panel) The RayBio® Cell-Based MAPK ELISA Kit was used for measuring the relative amount of MAPKs phosphorylation in cultured H9c2 cells. Each value represents the mean ± S.E.M, n = 3–4. ^#^*P* < 0.05 versus control; *P < 0.05, **P < 0.01 versus cells exposed to lysoPC alone.

To investigate the effects of baicalein on MAPK signaling, phosphorylated protein levels of JNK, p38, and ERK1/2 were measured in H9c2 cells by Western blotting (Figure 7a) and cell-based ELISA assay (Figure 7b). We found that stimulation of cell with lysoPC (10 μM) resulted in the expression of three MAP kinases-ERK1/2, JNK, and p38 phosphorylations. In addition, pretreatment with high concentrations of baicalein (1 to 10 μM) significantly blunted the lysoPC-induced phosphorylation of ERK1/2, JNK, and p38 (Figure 7a), then the activities of ERK1/2, JNK, and p38 cascades were blocked in the baicalein-related dose-dependent manner. Similarly results also found that baicalein attenuated lysoPC-induced activation of ERK1/2, JNK and p38 by ELISA quantification (Figure 7b).

## Discussion

To our knowledge, this is the first study to demonstrate that baicalein has composite cardioprotective effects against lysoPC-induced myocardial apoptosis. Here we showed that baicalein has significant effects on the Bcl-2/Bax ratio and on the levels of cytochrome *c*, caspase-3, and capspase-9 in a dose-dependent manner. Such results exhibited that baicalein possesses intense inhibitory effects on the lysoPC-induced mitochondrial-mediated apoptotic pathway.

It has been reported that the concentration of lysoPC in myocardium may increase in the setting of cardiac ischemia [12,13]. In addition, it is known that lysoPC could cause the Ca^2+^-influx in rat cardiomyocytes [14]. LysoPC accumulation in intracellular and/or interstitial space in cardiomyocytes may underlie as a mechanism for tachycardia and various arrhythmias during cardiac ischemia, which is usually accompanied by elevation of [Ca^2+^]i [2]. The exogenous lysoPC was also proven to induce myoischemia-like injury in in vitro study. As the increase of [Ca^2+^]i would cause irreversible injury of cardiomyocytes including reduction of cell-to-cell coupling, abnormal rhythmic activity and malignant intra-cardiac reentry, preservation of the level of [Ca^2+^]i within normal range is important for the normalization of cardiac function [2,13-15]. Therefore, a drug that can minimize the detrimental effects of lysoPC could attenuate cardiac dysfunction during myocardial ischemia. Here we show that baicalein attenuated cell death, apoptosis, and [Ca^2+^]i accumulation of H9c2 cells caused by lysoPC in a dose-dependent manner. Our results are in line with recent investigations showing that baicalein protects against balloon injury-induced intimal hyperplasia and aortic banding-induced cardiac hypertrophy and fibrosis [16,17]. Therefore, baicalein might have the therapeutic utility in the treatment of cardiovascular disease.

Overproduction of ROS may lead to cell death and apoptosis [6]. Recently studies strengthen the notion that ROS is an important mediator of lysoPC-induced cytotoxicity in both endothelial cells and VSMCs. Previous study suggested that ROS generated through NADH/NADPH oxidase are essential for the growth-promoting signals activated by lysoPC in VSMCs [18]. Oxidative stress promotes the apoptosis or death of cardiomyocytes and has been implicated in cardiovascular diseases, and the Bcl-2 family proteins are known as key regulators of the apoptotic response [19,20]. In present study, pretreatment of baicalein induced up-regulated the expression of Bcl-2 protein and down-regulated the production of Bax protein in H9c2 cells, resulting in a dramatic decrease in the Bax to Bcl-2 ratio, and also blocked the release of cytochrome *c* and activation of caspase-3 and caspase-9. The mechanisms of anti-apoptotic effects of Bcl-2 include: (1) act against the apoptotic gene of Bax; (2) inhibit the secretion of cytochrome *c* from the mitochondria to the cytoplasma, which are promoters of apoptosis; (3) block the trigger effects of cytochrome C on caspase cascades; and (4) induce the Bcl-2 proteins to carry the effects of anti-oxidation and stabilize the level of intracellular Ca^2+^. Actually, the ratio between the Bcl-2 and Bax helps to determine the susceptibility of cells to a death signal, and it has been suggested that the Bcl-2/Bax ratio may be more important than either promoter alone, in determining the apoptosis pathway [19-21].

MAPK family, one of downstream signal transduction pathways of ROS, is believed to function as integrators for cell growth, survival and apoptosis. In general, the p38 cascade mediates the apoptosis and the reactions of cytokines; the JNK cascade mediates inflammation, cell differentiation, and apoptosis; and the ERK cascade regulates cell differentiation and growth [22,23]. It is generally believed that phosphorylation of ERK1/2 can protect apoptosis of cardiomyocytes. However, there is some evidence suggesting that ERK1/2 also contributes to cell death of cardiomyocytes. For example, activation of ERK1/2 has a role in Bcl-2 family-mediated cell apoptosis caused by doxorubicin in cardiomyocytes [24]. In our study baicalein inhibited activations of all three MAPK induced by lysoPC, we speculate that the effect on ERK was offset by those beneficial effects on JNK and p38, resulting in net effects of preserving cell survival.

**Figure 8:**
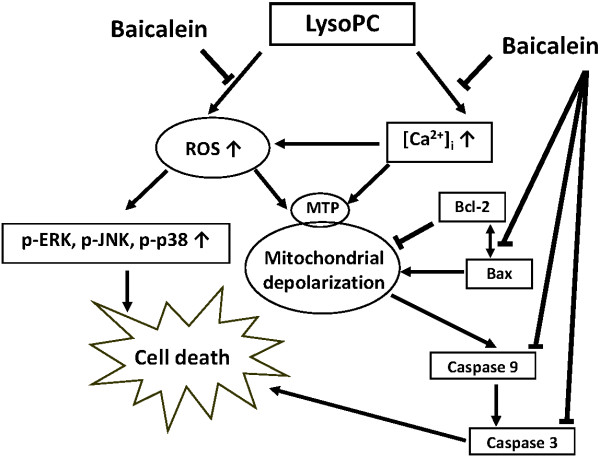
**Proposed mechanisms of the cardioprotective effects of baicalein on lysoPC-induced myocardial apoptosis according to results in the present study.** Baicalein can significantly inhibit myocardial apoptosis induced by lysoPC, at least in part, by reducing ROS production and calcium overload and blunting MAPK-mediated cascades.

The protective effects of baicalein are summarized and shown in Figure 8. In the study, we found baicalein not only inhibited the lysoPC-induced ROS production and intracellular Ca^2+^ accumulation, but also inhibited the three families of MAPK kinases (p38, JNK, and ERK) as well as the mitochondrial intrinsic apoptotic pathway.

There are two limitations of this study. First, the H9c2 cardiomyoblasts instead of cardiomyocytes were used in our study. The H9c2 cell line was derived from the embryonic rat heart, presenting similar properties of cardiomyocytes. However, future studies may be warranted to substantiate these findings in cardiomyocytes and myocardial tissue. Second, we decreased the highest dose of lysoPC from 50 μM to 10 μM in some experiments (MAPK and apoptosis). The reason is that high concentration of lysoPC (50 μM) could decrease of cell viability significantly, so that it was difficult to collect enough viable cells to perform these analysis.

## Conclusions

In summary, these novel results indicate that baicalein can protect H9c2 cells from lysoPC-induced cell death via reducing ROS production and anti-apoptosis. Such protective effects are possibly mediated through reduction of calcium overload, the mitochondrial intrinsic apoptotic pathway, and the MAPK signaling pathway. Thus, this study provides a cellular and molecular basis for baicalein in the treatment of lysoPC-induced cardiac injury.

## Competing interests

The authors declare that they have no competing interests.

## Authors’ contributions

H-MC, J-HH, C-CC, and J-LY participated in the design of the study; H-MC, S-FL, and L-YC carried out the experiments; H-MC, J-HH, T-JC, and J-LY analyzed the data, and wrote the paper. All authors read and approved the final manuscript.

## Pre-publication history

The pre-publication history for this paper can be accessed here:

http://www.biomedcentral.com/1472-6882/14/233/prepub
